# Femtosecond Laser-Induced Periodic Surface Structures on 2D Ti-Fe Multilayer Condensates

**DOI:** 10.3390/nano11020316

**Published:** 2021-01-27

**Authors:** Oleksandr V. Kuznietsov, George D. Tsibidis, Anatoliy V. Demchishin, Anatoliy A. Demchishin, Volodymyr Babizhetskyy, Ivan Saldan, Stefano Bellucci, Iaroslav Gnilitskyi

**Affiliations:** 1 NoviNano Lab LLC, Pasternaka 5, 79015 Lviv, Ukraine; oleksandr.kuznietsov22@gmail.com; 2 Department of Photonics, Lviv Polytechnic National University, 79013 Lviv, Ukraine; 3 Institute of Electronic Structure and Laser (IESL), Foundation for Research and Technology (FORTH), N. Plastira 100, Vassilika Vouton, 70013 Heraklion, Crete, Greece; tsibidis@iesl.forth.gr; 4 Frantsevich Institute for Problems in Materials Science of NASU, Krzhizhanovsky 3, 03142 Kyiv, Ukraine; demch@ipms.kiev.ua (A.V.D.); ntuu.kpi@ukr.net (A.A.D.); 5 Faculty of Chemistry, Ivan Franko National University of Lviv, Kyryla and Mefodia 6, 79005 Lviv, Ukraine; v.babizhetskyy@gmail.com (V.B.); saldanivan@gmail.com (I.S.); 6 Faculty of Science, P.J. Šafárik University in Košice, Šrobárova 2, 04154 Košice, Slovakia; 7 INFN-Laboratori Nazionali di Frascati, Via E. Fermi 54, 00044 Frascati, Italy; bellucci@lnf.infn.it

**Keywords:** vacuum-arc evaporation, titanium, iron, LIPSS, multilayer structures

## Abstract

2D Ti-Fe multilayer preparation has been attracting increased interest due to its ability to form intermetallic compounds between metallic titanium and metallic iron thin layers. In particular, the TiFe compound can absorb hydrogen gas at room temperature. We applied femtosecond laser pulses to heat Ti-Fe multilayer structures to promote the appearance of intermetallic compounds and generate surface nanostructuring. The surface pattern, known as Laser Induced Periodic Surface Structures (LIPSS), can accelerate the kinetics of chemical interaction between solid TiFe and gaseous hydrogen. The formation of LIPSS on Ti-Fe multilayered thin films were investigated using of scanning electron microscopy, photo-electron spectroscopy and X-ray diffraction. To explore the thermal response of the multiple layered structure and the mechanisms leading to surface patterning after irradiating the compound with single laser pulses, theoretical simulations were conducted to interpret the experimental observations.

## 1. Introduction

The TiFe intermetallic compound (IMC) is considered a promising hydrogen storage material, but this IMC requires initial activation to effectively absorb hydrogen gas [1,2,3,4,5,6]. Using high-pressure torsion (HPT), TiFe can reversibly absorb ~1.4–1.7 wt.% H2 in practice at room temperature without thermal activation [1]. Usually, TiFe is processed by three different routes: annealing, plastic deformation using groove rolling, and severe plastic deformation using HPT [2]. Effective activation of air-exposed TiFe alloy might be performed mechanically using cold rolling [3,4] or ball milling [3,5,6]. These procedures help to restore the hydrogenation capability of TiFe. Because of the low price of production and activation of TiFe, it can be used in hydrogen storage tanks, fuel cells and secondary batteries. Recently, the operation of a bench-scale stationary hydrogen energy system comprising photovoltaic panels, a water electrolyzer and a TiFe-based tank was tested in practice [7]. Thin films of pure TiFe can be used as hydrogen sensors at room temperature, but in order to charge/discharge them faster, a more developed surface structure is needed. The nanostructuring of solid surfaces may be a reasonable way of accelerating the kinetics of their interaction with gaseous hydrogen.

Fabricating a surface pattern that can be used for the aforementioned purpose can be achieved through numerous methods including multi-beam interference-based techniques and Laser Induced Periodic Surface Structures (LIPSS). LIPSS is a potentially strong method, as it is a single-step, maskless and optical patterning technique. This method of surface texturizing has been realized on metals [8], semiconductors [9], dielectric surfaces [10] and polymers [11]. Moreover, LIPSS were used in numerous applications including solar cells [12], plasmonics [13], colorizing metals [14,15], wettability [16] and tribology [17]. Recently, advanced techniques for generating highly-regular LIPSS (HR-LIPSS) have been developed to create high-quality periodic nanostructures over large area [18]. Nanolayers with a high level of regularity were obtained over a large surface area with a single-step maskless process and industrially accepted speed production. The use of the HR-LIPSS approach allows laser self-organized periodic structures with practical applications to be obtained, as they are sensitive to the optical, mechanical, adhesive and wetting properties of the surface.

The nanostructured pattern (LIPSS) of the Ti-Fe system was obtained by using femtosecond laser pulses to create a functional profile. In order to interpret the physical mechanisms that account for the surface modification, simulations were performed to highlight the thermal response of the femtosecond laser pulses during the LIPSS formation on multilayers Ti-/Fe system. The theoretical predictions, based on the 1D two-temperature model (TTM) simulations, were in good agreement with the experimental results.

## 2. Experimental Part

### 2.1. Preparation of Ti-Fe Multilayer

Made in a vacuum, thick condensates of the Ti/Fe system composed of numerous layered heterogeneous layers of the separate metals were obtained using the device “Bulat-3T” (Kharkiv, Ukraine) through the method of consecutive condensation from unfiltered plasma flows, which were respectively generated by stationary cathode-type vacuum arcs under conditions of constant ion bombardment of the formed condensates by applying an electric negative potential to the substrates [19]. The distance between the end-type cathodes and the substrates was 125 mm. Steel plates were used as a substrate made of X12Cr17 coiled steel with a size of 100 nm × 100 nm × 0.3 mm. Cathodes with a diameter of 64 mm were obtained by machining ingots of Ø70 mm from pure Ti and Fe, melted by electron beam remelting in a vacuum of 1 × 10^−4^ mm Hg. The condensation duration of each sublayer was 10, 20, 30, 40, 50 s, which made it possible to obtain condensates while modulating the multilayer structure in the range of 125–620 nm. The total thickness of the multilayer compositions was 60–80 microns. A negative potential of 180V was applied to the substrates. The metal deposition rate was 0.8–1.2 μm/min. The total duration of the deposition of multilayer compositions is 60 min. The arc current was tantamount to 100A and 80A for cathodes of Ti and Fe, respectively. The value of the working pressure in the installation chamber was 3–4 × 10^−3^ mm Hg. The vacuum annealing of condensates without separation from the substrates was carried out in this unit at a temperature of 650–700 °C by bombarding their surfaces with argon ions for 30 min as a way of applying a negative potential of 1 kV to the substrates and a pressure of 3–4 × 10^−3^ mm Hg in the chamber. The structure of the coatings was studied using a JEOL 733 scanning electron microscope (Tokyo, Japan). The phase composition of multilayer condensates was determined using X-ray diffraction analysis. XRD analysis was performed using Cu K_α_ radiation. The microhardness of the condensates was measured using a PMT-3 microhardness meter at a load of 50 g on transverse sections.

### 2.2. Processing with Ultrashort Laser

**Figure 1:**
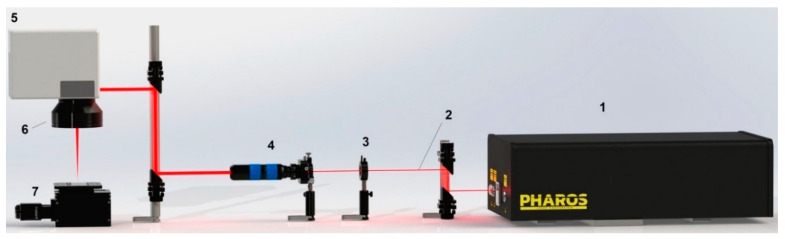
Laser setup for generation LIPSS: **1**—femtosecond laser “Pharos”, **2**—laser beam, **3**—HWP (half wave plate), **4**—expander, **5**—galvoscaner, **6**—F-theta lens, **7**—z-axis motorized stage.

Heat-treated samples with a thickness of 60–80 μm were irradiated with a Yb: KGW laser (model Pharos 20 W, LightConversion, Vilnius, Lithuania) with 213 fs pulses and a spectrum width of 15 nm (Figure 1). The pulse repetition rate was 600 kHz. The fluence was in the range of 0.5 J/cm^2^. Laser processing of the samples was carried out in air at room temperature in the regime of scanning a laser beam over their surface. The average power output was 20 W. The laser setup was equipped with lenses with a focal length of 56 mm, which ensured that a laser spot with a diameter of 10.4 μm was obtained at ℓ/e^2^ intensity. The size of the lateral displacement of the laser beam was adjustable from 2 to 4 microns. Laser installation allows one to scan the surface of the samples with a linear speed of 0.5 m/s and an equivalent productivity of about 900 mm^2^/min. The topography of the laser-treated sample surface was studied using an FEI Nova Nano SEM 450 electron microscope (Hillsboro, OR, USA).

### 2.3. X-ray Photoelectron Spectroscopy (XPS)

XPS data were recorded using SPECS spectrometer (Berlin, Germany) with a PHOIBOS hemispherical energy analyzer and monochromatic Al Kα X-ray irradiation (*hν* = 1486.74 eV, 350 W). All spectra were calibrated using the C 1s main peak located at a binding energy (BE) of 284.6 eV. The atomic concentrations of the elements were calculated taking into account corresponding relative sensitivity factors in CasaXPS software program.

### 2.4. X-ray Diffraction Analysis (XRD)

XRD patterns were recorded using a Rigaku diffractometer, equipped with a Miniflex goniometer and an X-ray source with Cu Kα radiation, at λ = 1.5418 A, 30 kV, and 15 mA. The samples were scanned in the 1–80°, 2θ range with a scan rate of 0.02°/s. Diffraction patterns were assigned using Joint Committee on Powder Diffraction Standards (JCPDS) cards supplied by the International Centre for Diffraction Database (ICDD).

### 2.5. Scanning Electron Microscope (SEM) and Focused Ion Beam (FIB) Characterization

The surface morphology of the untreated samples and those treated with ultrashort laser pulses was studied using Fa scanning electron microscope FEI Nova Nano SEM 450 with Bruker QUANTAX-200 X-EDS (Billerica, MA, USA). The cross-sections and the corresponding images were obtained by means of an FEI Strata 235 M dual beam system (Hillsboro, OR, USA). The system combines a Focused Ion Beam (FIB) equipped with a Ga Liquid Metal Ion Source (LMIS). The perpendicular cross-sections of the Ti/Fe-coated surfaces were obtained using an FIB (E-beam = 30 keV) for milling, setting 1 nA as the ion beam current for milling and 300 pA for the final polishing. In order to protect the topmost material of the coating from the ion mixing effect, the surface was protected by a thin Pt layer (Pt shield) deposited in situ using Ion Beam.

## 3. Research Results and Their Discussion

### 3.1. SEM Observation

**Figure 2:**
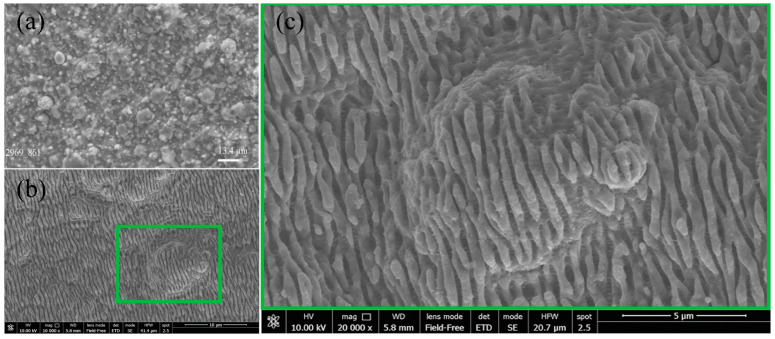
SEM observation of the surface of Ti/Fe (**a**) after vacuum arc condensation and after LIPSS formation on their surface (**b**) and its magnified view (**c**).

SEM shows a typical surface morphology of metallic Ti or Fe layers after vacuum arc condensation after arc melting. The surface is chaotically covered with many inhomogeneous particles left after melting (Figure 2a). However, laser treatment with LIPSS formation leads to the structuring of the surface by creating periodic longitudinal ripples on Ti/Fe systems when the Ti layer is on the top (Figure 2b,c). LIPSS were successfully generated over a large area of 1 cm^2^. Despite a lot of bifurcations that were created as a collateral effect of vacuum-arc deposition, LIPSS were homogeneously obtained over all large areas, which can be attributed to the HR-LIPSS method [20]. The quality of LIPSS was measured by a descriptor of the regularity of the periodic structures, DLOA (Dispersion of the LIPSS orientation angle), which is presented in-depth in [20]. DLOA was measured at 18.5°, thus certifying that periodic structures have a good quality despite of the numerous defects on the surface. The period of periodic structures is in the range of 650–750 nm, while the direction of the periodic structures is perpendicular to the laser polarization, which suggests an ablation mechanism for LIPSS formation.

**Figure 3:**
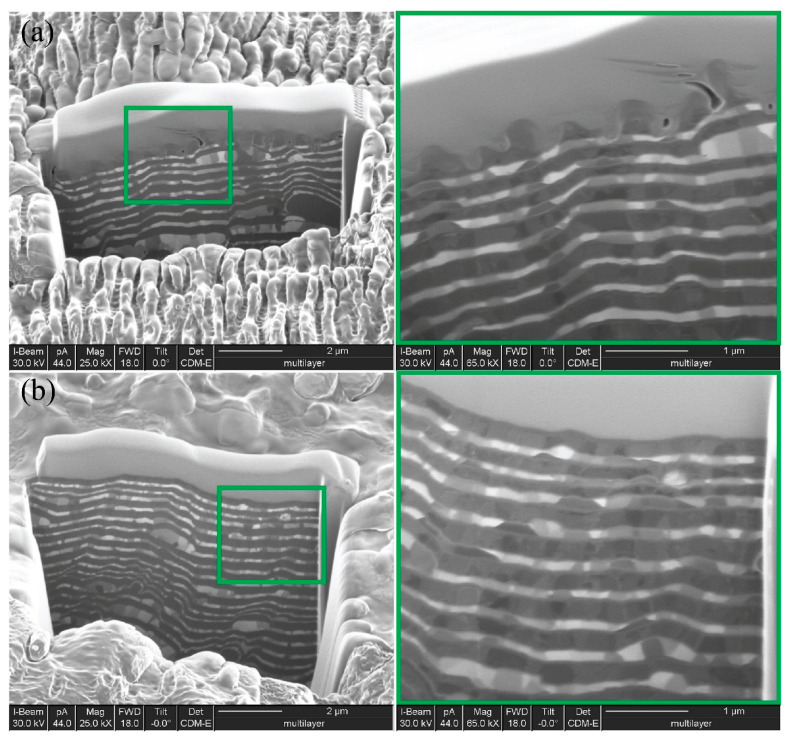
SEM micrographs of the vacuum-arc condensed Ti-Fe multilayer with layer formation (**b**) and after LIPSS formation (**a**) made by shot by gallium ion beam.

The focused ion beam (FIB) technique demonstrates the cross-sections of Ti-Fe bilayered structures, non-irradiated as well as irradiated by laser pulses (Figure 3). This approach revealed the periodicity of metallic titanium and iron layers, suggesting a “sandwich-type” structure. The thickness of a separate layer was in the range of 90–100 nm; moreover, this value was directly proportional to the deposition time. The cross-section image on the patterned area (Figure 3b) shows that each ripple is much deeper than the thickness of the Ti or Fe layers, and consists of at least two altered layers. The width of the columnar crystallites, which was elongated in the direction of the steam flow, increased with the respective increase of the substrate temperature. It was well visible that the periodic metal layers were not completely uniform in color. That is, in addition to light and dark areas, there were also mixed shades. This suggested an alloying between two metallic layers that may occur at temperatures even lower than those in the case of bulk metals using the Ti-Fe binary phase diagram; two intermetallic compounds, Fe_2_Ti and FeTi, may exist in the narrow regions of two components [21]. Thus, in subnano-multilayer condensates of metallic titanium and iron, the formation of the TiFe intermetallic compound at the layer interface might be observed in conformity to the Ti-Fe phase diagram.

### 3.2. XPS Analysis

The surface of the Ti-Fe multilayers was analyzed by XPS. The C 1s, Fe 2p, O 1s, Ti 2p, N 1s, and Ca 2p photoelectron core level spectra of the detected elements were monitored (Table 1). The obtained XPS signals were assigned in accordance with the XPS database [22]. Along with amorphous carbon on the surface, some carbonates (C 1s at ~288.2 eV) were detected. In total, ~44 and ~35 at.% of Carbon was detected by XPS for the as-prepared and laser-treated Ti-Fe multilayer samples. These values might be considered to be surface contamination at the metallic surface [23]. The signal of O 1s demonstrates two intense peaks at 529.7 eV and 531.4 eV. The first peak suggests surface oxides on both TiO_2_ and Fe_2_O_3_, and the second one might be associated with oxygen in the surface carbonates. The total amount of oxygen was ~45 at.%. In addition to Fe III oxide, metallic iron was also found on the surface. The Fe 2p_3/2_ core level in XPS spectra reveals two peaks at 706.4 and 710.4 eV related to Fe^0^ and Fe^3+^ chemical states, respectively. The amount of Fe_2_O_3_ was higher than metallic Fe in one order. Similar to iron, titanium was also found in two chemical states: Ti^0^ (Ti 2p_3/2_ at ~453.5 eV and Ti^4+^ (Ti 2p_3/2_ at ~458.4 eV) where metallic titanium is dominated by TiO_2_. Calcium in the form of Ca^2+^ (Ca 2p_3/2_ at ~347.2 eV was also detected by XPS. Most probably, this is because of typical surface contamination CaCO_3_ XPS signals at 400.5 and 396.4 eV might be attributed to the N 1s signal in organic compounds with C–N bonds and metal nitrides, respectively.

Both the as-prepared and laser-treated Ti-Fe multilayer samples were contaminated by carbon, oxygen and nitrogen, which is very common for as-prepared or laser-treated surfaces in open air. In addition, XPS data suggested a residual CaCO_3_ on the surfaces.

### 3.3. XRD Analysis

**Figure 4:**
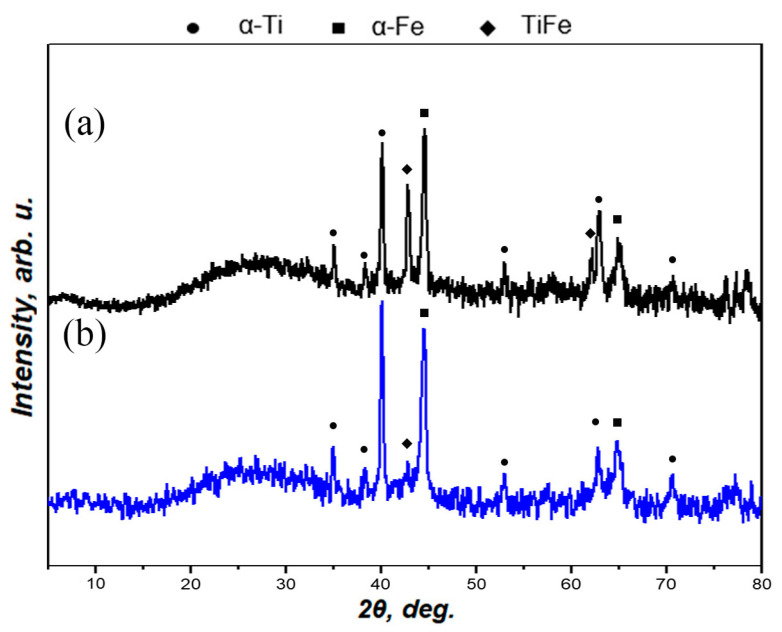
XRD patterns for the laser-treated (**a**) and as-prepared (**b**) Ti-Fe multilayer samples.

XRD patterns for as-prepared and laser-treated Ti-Fe multilayer samples are shown in Figure 4. Both samples had an amorphous halo in range of 20–35°, and there was only one difference in the peak intensity of the TiFe intermetallic compound. After heat treatment of the Ti-Fe multilayer, the intermetallic compound TiFe was synthesized. The laser treatment applied to the surface resulted in a partial removal of the surface layer, since te surface of the prepared Ti-Fe multilayer samples was thoroughly “scratched”. This is probably the reason why the TiFe phase, which appeared after heat treatment located between the metallic titanium and the iron layer, became more pronounced in the XRD pattern. In other words, the laser treatment of the Ti-Fe multilayer might be considered a technical tool for both surface structuring (Figure 2b,c) and opening the way to TiFe thin layers (Figure 4).

### 3.4. AFM Analysis

Atomic force microscope (AFM) analysis of the surface roughness was done for five randomly selected parts of the surface (Table 2). It is clear that all experimental values of surface area for the laser treated Ti-Fe multilayer structure were higher than those for the as-prepared Ti-Fe multilayer. This suggests that LIPSS are developing surface of Ti-Fe multilayer surface.

## 4. Theoretical Model-Simulation Procedure

To interpret the experimental observations, simulations were conducted to explore the thermal response of the multiple layered structure 15x(Ti/Fe) after irradiation with single laser pulses with a pulse duration of *τ_p_* = 213 fs and a wavelength *λ_L_* = 1030 nm. An 1D-Two Temperature Model (TTM) [24] was used to describe the relaxation process following electron excitation due to laser heating through the following equations [25,26]:

(1) $$\begin{array}{l} C_{e}^{\left(i\right)}\frac{\partial T_{e}^{\left(i\right)}}{\partial t}=\frac{\partial }{\partial z}\left(k_{e}^{\left(i\right)}\frac{\partial T_{e}^{\left(i\right)}}{\partial z}\right)-G_{eL}^{\left(i\right)}\left(T_{e}^{\left(i\right)}-T_{L}^{\left(i\right)}\right)+S^{\left(i\right)}(z,t)\;\;\;\;\;\;[S^{\left(i\right)}=0,\;\mathrm{for}\;i>1]\\ C_{L}^{\left(i\right)}\frac{\partial T_{L}^{\left(i\right)}}{\partial t}=\frac{\partial }{\partial z}\left(k_{L}^{\left(i\right)}\frac{\partial T_{L}^{\left(i\right)}}{\partial z}\right)+G_{eL}^{\left(i\right)}\left(T_{e}^{\left(i\right)}-T_{L}^{\left(i\right)}\right) \end{array}$$

(2) $$S^{\left(1\right)}(z,t)=\frac{\alpha (1-R-T)\sqrt{4\log2}F}{\sqrt{\pi }\tau_{p}}\exp\left(-4\log2{\left(\frac{t-3\tau_{p}}{\tau_{p}}\right)}^{2}\right)\exp(-\alpha z)$$

In Equations (1) and (2), $T_{e}^{\left(i\right)}$($T_{L}^{\left(i\right)}$) stands for the electron (lattice) temperature of layer *i* (*i* = 1, 3, 5... 2n−1 for Ti layer, *i* = 2, 4, 6… 2n for Fe layer, for *n* = 300 Ti/Fe layers for a multi-layered thickness equal to 60μm; each layer has a thickness equal to 100 nm). The thermophysical properties of the materials, such as electron and lattice heat capacity, ($C_{e}^{\left(i\right)}$,$C_{L}^{\left(i\right)}$), electron and lattice heat conductivity ($k_{e}^{\left(i\right)}\equiv k_{e0}^{\left(i\right)}\left(B^{\left(i\right)}T_{L}^{\left(i\right)}/\left(A^{\left(i\right)}{\left(T_{e}^{\left(i\right)}\right)}^{2}+B^{\left(i\right)}T_{L}^{\left(i\right)}\right)\right)$, $k_{L}^{\left(i\right)}\sim 0.01k_{e}^{\left(i\right)}$), and electron-phonon coupling strengths ($G_{eL}^{\left(i\right)}$), and the model parameters used in the simulations are listed in Table 3.

While Equation (2) provides the general expression of the form of the source term due to material heating with a pulsed laser that includes the absorption coefficient *α*, the reflectivity *R* and the transmission coefficient *T* of the material, the Transfer Matrix Method was used to compute the optical properties of the top layer (Ti) after irradiation with pulsed laser of 1030 nm by taking into account the presence of the rest of the thin layers. Calculations yield *α =* 4 × 10^5^ cm^−1^ [32], *T* ≅ 0, *R* = 0.54, indicating that most of the energy will be absorbed in the first layer (Ti), while the transmitted part of the laser energy into the second layer (Fe) is essentially zero, due also to the thickness of the layer (100 nm), and it is not sufficiently high to excite the electrons in the rest of the layers (especially the second layer) and produce meaningful results. This argument justifies the use of a source term to describe laser heating only of the first layer and it is assumed that laser energy is not transmitted into the next layers. In principle, only heat transfer between the top few layers is expected, while optical excitation of the rest of the layers (except from the top one) is negligible.

Equations (1) and (2) are solved by using an iterative Crank–Nicolson scheme based on a finite-difference method. For the initial conditions, we chose thermal equilibrium at *T_e_(z,t = 0) = T_L_(z,t = 0) =* 300 K. Adiabatic boundary conditions are considered on the surface (at *z* = 0, $k_{e}^{\left(\mathrm{Ti}\right)}\frac{\partial T_{e}^{\left(\mathrm{Ti}\right)}}{\partial z}$=$k_{L}^{\left(\mathrm{Ti}\right)}\frac{\partial T_{L}^{\left(\mathrm{Ti}\right)}}{\partial z}$=0) and the back of the complex structure. Furthermore, at the interface between the two layers, the following conditions are applied: $T_{L}^{\left(\mathrm{Ti}\right)}=T_{L}^{\left(\mathrm{Fe}\right)}$,$T_{e}^{\left(\mathrm{Ti}\right)}=T_{e}^{\left(\mathrm{Fe}\right)}$, $k_{L}^{\left(\mathrm{Ti}\right)}\frac{\partial T_{L}^{\left(\mathrm{Ti}\right)}}{\partial z}=k_{L}^{\left(\mathrm{Fe}\right)}\frac{\partial T_{L}^{\left(\mathrm{Fe}\right)}}{\partial z}$, $k_{e}^{\left(\mathrm{Ti}\right)}\frac{\partial T_{e}^{\left(\mathrm{Ti}\right)}}{\partial z}=k_{e}^{\left(\mathrm{Fe}\right)}\frac{\partial T_{e}^{\left(\mathrm{Fe}\right)}}{\partial z}$. The evaluation of the thermal response of the material following irradiation with single pulses was performed through the correlation of the simulation results with the measured ablation. As noted in previous reports, ablation may be associated with the lattice temperature exceeding the condition (~0.90 *T_critical_* where *T_critical_* is the critical point temperature [33,34,35]). Another criterion that is also usually employed is the boiling temperature of the material, *T_boiling_* (i.e., the region of the material that is characterized with lattice temperatures higher than *T_boiling_* is removed [29]). In this work, the latter condition is used, as the experimental results indicate that there is ablation, while theoretical calculations do not yield lattice temperatures (~0.90 *T_critical_*).

## 5. Discussion

Despite a lot of bifurcations being created as a collateral effect of vacuum-arc deposition, the LIPSS were quite regular over all of the large areas, which can be attributed to the HR-LIPSS method [20]. Moreover, as shown by AFM, LIPSS increase the relative surface area by 9% with respect to the untreated sample, which might translate into a positive impact on kinetics for chemical interaction between solid TiFe and gaseous hydrogen. We expect that the surface properties are crucial for the acceleration of hydrogen gas adsorption on the solid surface that, together with hydrogen atom diffusion, might be a limiting stage for the hydrogenation process.

Because the laser treatment occurred in open air, the surfaces of the Ti-Fe multilayer samples were contaminated by amorphous carbon, CaCO_3_, TiO_2_ and Fe_2_O_3_. XPS experiments confirmed these surface contaminations, which are typical for a solid surface. AFM and XPS results suggested that because of the LIPSS treatment, the surface area of the Ti-Fe multilayers was increased; however, their surface cleanness was not affected. In practice, the laser treatment resulted in solid surface structuring and opened the way to the TiFe compound located under the surface of the prepared Ti-Fe multilayer samples. Experimental results obtained by SEM and XRD suggested that for the prepared Ti-Fe multilayers, alloying between metallic titanium and metallic iron resulted in the appearance of a TiFe compound. Usually, production of TiFe as ingot might be carried out directly by arc melting of metallic titanium and iron through the following reaction:

Ti + Fe = TiFe + 40.6 kJ.

At the same time, chemical methods including calciothermic reduction of TiO_2_ also enable the production of TiFe, but in the form of a fine powder [36]:

TiO_2_ +Fe + 2Ca = TiFe + 2CaO + 365.7 kJ.

The TiFe compound belongs to the hydrogen storage materials, in which the hydride has a similar crystal structure as the starting compound, although lattice expansion takes place during hydrogenation. More specifically, the crystal structure of the hydrogenated TiFe might be considered the derivative of that of the TiFe parent compound. The hydride stability is related to the strength of the chemical bond between metal atoms in a polyhedron in which H atoms are located. Therefore, hydrogen pressure is correlated with the ratios of the bond order between the metal atoms in the polyhedron [37,38]. On the other hand, the TiFe intermetallic compound has a CsCl-type structure, where expansion and contraction of the interatomic distances between Ti atoms during hydrogenation are 0.092 and 0.014 nm, respectively, while no changes are observed between Ti and Fe atoms. Thus, the H atom can occupy the interstitial site that is the Ti_2_Fe_4_ octahedron [39], resulting in a formula of hydride of TiFeH_1.94_ with a calculated hydrogen content of 1.9 wt.% H_2_.

**Figure 5:**
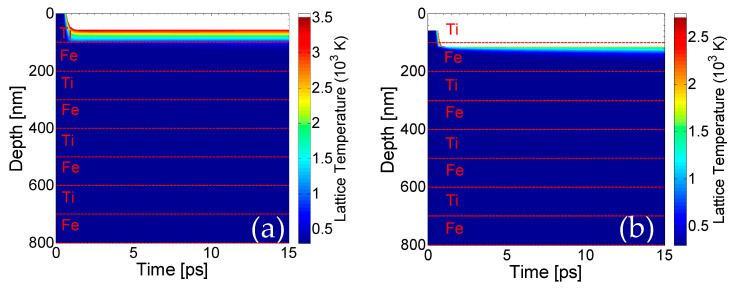

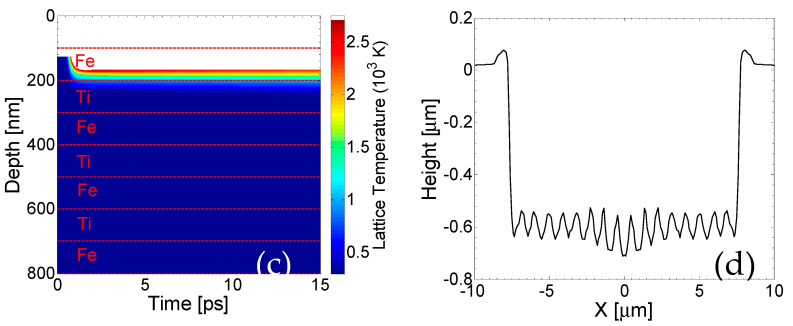
Lattice temperature field evolution in depth (in the center of the Gaussian spot) perpendicular to the surface of the sample after one pulse (**a**), two pulses (**b**) and three pulses (**c**) (white region indicates material removal, horizontal dashed line show the borders of the films); (**d**) periodic pattern after fourteen pulses (laser polarization is vertical to the figure plane).

Theoretical calculations of the lattice temperatures based on the scheme described above yield a spatio-temporal evolution that is illustrated in Figure 5 (in the center of the Gaussian spot). Our simulations and comparison with experimental observations indicate that nearly half of the first layer (Ti) is removed at fluence *F* = 0.5 J/cm^2^ (Figure 5a) and a second pulse is required to remove the Ti top layer completely (Figure 5b) This value corresponds to fluence that is sufficient to raise the temperature of the upper layer above *T_boiling_*; therefore, the boiling temperature is regarded as a reliable ablation threshold criterion. It is noted that for each pulse, only the first pair of Ti/Fe layers below the upper surface thermally respond to the heat transfer. Thus, for the sake of simplicity, only a region with a small number of layers is shown to describe the affected zone.

It is important to note that due to the fact that lattice temperatures on the second layer (Fe) are lower than the boiling temperature for Ti, no material is predicted to be removed from the second layer. On the other hand, it is evident that the lattice temperature attained from a large part of the second layer (Fe) for *F* = 0.5 mJ/cm^2^ is above the melting point of the material. Finally, Figure 5c illustrates the effect of the third pulse, showing that due to the small heat capacity, large conductivity and lower boiling temperature, the energy of the laser propagates inside the Fe-layer; however, it is only sufficient to remove part of the Fe-layer, while another portion of the rest undergoes a phase transition, as the lattice temperatures lie between the melting point and the boiling temperature. The value for the reflectivity and absorption coefficient used to simulate energy absorption for Fe are 0.6173 and *α =* 4 × 10^5^ cm^−1^, respectively, when Fe is on the upper surface. The above description indicates that fluid dynamics and resolidification processes are expected to further modify the surface profile of the assembly. Therefore, appropriate phase change-related corrections need to be incorporated into the model for a more accurate description of the surface modification processes and determination of the morphological changes. A thorough approach requires the inclusion of Navier–Stokes equations (to describe fluid dynamics) and relevant equations to account for evaporation [32,35,36,37]. The fact that mass displacement and mass removal is expected to lead to a corrugated profile means that appropriate conditions for surface plasmon excitation can be easily satisfied (for a detailed description, see Refs [32,40,41,42]). Thus, a periodic pattern can be produced, which is also confirmed by the experimental results [43,44]. This occurs irrespective of which material is on top (Fe or Ti). Although most studies have been centered on the investigation of surface plasmon (SP) excitation in dielectric–metal–dielectric multi-layered films and potential coupling of the excited SP on the dielectric–metal and metal–dielectric films in the periodicity of the periodic structures [45,46], to the best of our knowledge, no previous study has been conducted for air/multi-layered (metallic) heterostructures. Certainly, as there is no alteration of dielectric/metallic films to sustain excitation of bound SP, only SP on the top layer should be expected to have a periodicity the size of the laser wavelength (~1 μm). Nevertheless, it is known that the SP wavelength decreases at increasing energy doses [47]. The aforementioned simulations predict a sufficient ablation that induces an enhanced depth in the irradiated heterostructure, and therefore, SP wavelength should decrease. Although a consistent approach based on the employment of Finite Difference Time domain codes to solve Maxwell’s equations is expected to provide a precise correlation of the ripple periodicities as a function of the produced depth (see Ref. [48] and references therein), an approximate methodology is used (see Ref. [48]). More specifically, for fourteen pulses, a SP wavelength equal to 920 nm is predicted, which yields a similar ripple periodicity on Fe illustrated in Figure 5d. Experimental measurements performed for 12.5 pulses give a ripple periodicity in the range of 650–750 nm. Certainly, a theoretical investigation assuming the surface patterning effect following a larger number of pulses could be conducted for a quantitative comparison of the ripple periodicities; however, the predominant aim of this work was focused on the thermal response of the material and the ablation-assisted modification of the surface layers. The reason why fourteen pulses were used in the simulations was to clearly illustrate both the damage induced on the complex (removal of layers) and the periodicity of the rippled zone. It is evident that there is a discrepancy between the experimentally measured value and the theoretical prediction that requires more investigation; however, the aim of the current work was to highlight the step-by-step removal of the metallic layers. Thus, the development of an improved theoretical model that yields a better agreement with the experimental observations could be the subject of a future work.

## 6. Conclusions

The intermetallic compound TiFe was formed between multilayers made of metallic titanium and metallic iron and was detected using both XPS and XRD. The LIPSS method was applied, which generated homogeneous ripples over a large area, even on non-polished surfaces densely covered with numerous defects and bifurcations. Because of the generation of a more developed Ti-Fe multilayer surface, the absorption/desorption of gaseous hydrogen was accelerated. The thermal effects resulting from the irradiation of the compound were simulated to predict the affected region as well as to predict the periodicity of the surface pattern.

Laser-treated Ti-Fe multilayers will be tested with respect to hydrogen gas absorption and optimized as a hydrogen sensor in future publications.

## Figures and Tables

**Table 1:** Surface element analysis evaluated with XPS.

Sample	Ti, at %		Fe, at %		C, at %			O, at %		Ca, at %	N, at %	
Ti-Fe as prepared	2.3		8.2		43.5			44.4		0.3	2.9 C-N	
	2.0 TiO_2_	0.3 Ti^0^	7.7 F_2_O_3_	0.5 Fe^0^	31.6 other C	11.9 CO_3_^2−^		23.2 oxide	21.2 CO_3_^2−^	Ca^2+^		
Ti-Fe laser treated	14.6		4.5		34.6			44.9		0.2	1.2	
	11.9 TiO_2_	2.7 Ti^0^	4.0 F_2_O_3_	0.5 Fe^0^	28.6 other C	2.1 carbide	3.9 CO_3_^2−^	25.5 oxide	19.4 CO_3_^2−^	Ca^2+^	1.1 C-N	0.1 nitride

**Table 2:** Values of the surface area obtained by AFM respect to a projection area of 95.36 μm^2^.

Sample	Surface Area of the Five Selected Parts of the Surface, μm^2^.					
	1	2	3	4	5	Average
Ti-Fe laser treated	136.691	134.578	132.351	132.057	136.347	134.405
Ti-Fe as prepared	123.707	123.836	123.513	122.829	122.577	123.292

**Table 3:** Simulation parameters for Ti and Fe.

Parameter	Ti	Fe
*G_eL_* [Wm^−3^K^−1^]	Fitting [26,27]	Fitting [28]
*C_e_* [Jm^−3^K^−1^]	Fitting [26,27]	Fitting [28]
*C_L_* [Jm^−3^K^−2^]	2.3521 × 10^6^ [29]	3.675 × 10^6^ [30]
*k_e0_* [Jm^−1^s^−1^K^−1^]	21.9 [29]	46.6 [30]
*T_melting_* [K]	1941 [29]	1811 [30]
*T_boiling_* [K]	3560 [29]	2750 [30]
*T_critical_* [K]	15500 [31]	8500 [30]
*A* [s^−1^ K^−2^]	Fitting [26,27]	0.98 × 10^7^ [30]
*B* [s^−1^ K^−1^]	Fitting [26,27]	2.8 × 10^11^ [30]
